# Identification of a Rho-Dependent Termination Site *In Vivo* Using Synthetic Small RNA

**DOI:** 10.1128/spectrum.03950-22

**Published:** 2023-01-18

**Authors:** Xun Wang, Monford Paul Abishek N, Heung Jin Jeon, Jin He, Heon M. Lim

**Affiliations:** a State Key Laboratory of Agricultural Microbiology, College of Life Science and Technology, Huazhong Agricultural University, Wuhan, Hubei, People’s Republic of China; b Department of Biological Sciences, College of Biological Sciences and Biotechnology, Chungnam National University, Daejeon, Republic of Korea; c Infection Control Convergence Research Center, Chungnam National University College of Medicine, Daejeon, Republic of Korea; Centre National de la Recherche Scientifique, Aix-Marseille Université

**Keywords:** synthetic sRNA, Rho-dependent termination, RNase III, RNA processing, exonuclease

## Abstract

Rho promotes Rho-dependent termination (RDT) at the Rho-dependent terminator, producing a variable-length region without secondary structure at the 3′ end of mRNA. Determining the exact RDT site *in vivo* is challenging, because the 3′ end of mRNA is rapidly removed after RDT by 3′-to-5′ exonuclease processing. Here, we applied synthetic small RNA (sysRNA) to identify the RDT region *in vivo* by exploiting its complementary base-pairing ability to target mRNA. Through the combined analyses of rapid amplification of cDNA 3′ ends, primer extension, and capillary electrophoresis, we could precisely map and quantify mRNA 3′ ends. We found that complementary double-stranded RNA (dsRNA) formed between sysRNA and mRNA was efficiently cleaved by RNase III in the middle of the dsRNA region. The formation of dsRNA appeared to protect the cleaved RNA 3′ ends from rapid degradation by 3′-to-5′ exonuclease, thereby stabilizing the mRNA 3′ end. We further verified that the signal intensity at the 3′ end was positively correlated with the amount of mRNA. By constructing a series of sysRNAs with close target sites and comparing the difference in signal intensity at the 3′ end of wild-type and Rho-impaired strains, we finally identified a region of increased mRNA expression within the 21-bp range, which was determined as the RDT region. Our results demonstrated the ability to use sysRNA as a novel tool to identify RDT regions *in vivo* and expand the range of applications of sysRNA.

**IMPORTANCE** sysRNA, which was formerly widely employed, has steadily lost popularity as more novel techniques for suppressing gene expression come into existence because of issues such as unstable inhibition effect and low inhibition efficiency. However, it remains an interesting topic as a regulatory tool due to its ease of design and low metabolic burden on cells. Here, for the first time, we discovered a new method to identify RDT regions *in vivo* using sysRNA. This new feature is important because since the discovery of the Rho protein in 1969, specific identification of RDT sites *in vivo* has been difficult due to the rapid processing of RNA 3′ ends by exonucleases, and sysRNA might provide a new approach to address this challenge.

## INTRODUCTION

Bacteria utilize transcription terminators to terminate transcription, which are classified in the categories of Rho-independent termination (RIT) and Rho-dependent termination (RDT) according to sequence properties and regulatory mechanisms (1). The Rho-independent terminator consists of two main modules: a guanine- and cytosine-rich RNA stem-loop structure and a downstream 7- or 8-bp uridine-rich sequence called the U tract. When the U tract is transcribed, it stalls RNA polymerase (RNAP) long enough to allow stem-loop formation, which disrupts the transcription complex, resulting in the termination of transcription in the U tract (2–4). Precise localization of RIT sites *in vivo* is not difficult, since the sequence features required for RIT function are well defined and transcription termination occurs in U-shaped tracts (5, 6).

Rho is a ring-shaped homohexameric protein with RNA-dependent ATPase activity (7), which enables Rho to translocate along nascent transcripts and mediate the release of RNA transcripts from the transcription complex paused at the termination region (8–10). The Rho-dependent terminator also contains two main modules: a cytosine-rich and guanine-poor Rho-binding site (C-rich region) that lacks a secondary structure, and a termination region downstream of the C-rich region (11). Computational prediction of Rho-dependent terminators is not straightforward due to the complex and poorly defined sequence features required for the Rho function. Nadiras et al. and Di Salvo et al. constructed prediction models of Rho-dependent terminators (12, 13). However, experiments by Chhakchhuak and Sen confirmed that the accuracy of the model needs to be improved (14). Therefore, experimental methods to identify RDT loci are highly desired.

There are two main methods for detecting terminators *in vivo*, depending on whether the terminator is detected on the plasmid or the chromosome. The first method is to construct a series of plasmids, clone the terminator upstream of the reporter gene, and determine the strength of the terminator by comparing the expression level of the reporter gene. Using this method, Mott et al. and Hart and Roberts identified the Rho-dependent terminator trp t′ of the tryptophan operon and the Rho-dependent terminator tR1 of the λ phage, respectively (15, 16). In Escherichia coli, transcription is coupled to translation, and the translating ribosomes repress RDT by sterically blocking Rho from entering the nascent transcripts (17). Uncoupling of translation and transcription allows Rho to bind the C-rich region and induce transcription termination (18). However, the terminators trp t′ and tR1 located in the intergenic region are not translated. When the terminator is cloned into the plasmid, the region upstream of the reporter gene is also not translated. Therefore, terminators are untranslated both on the plasmids and on the chromosomes, and they are not subject to translational repression. On the other hand, two potential Rho-dependent terminators, *tiZ1* and *tiZ2*, located in the intragenic region of *lacZ* are inactive when *lacZ* mRNA is translated (18, 19). When a terminator is cloned from a chromosome to a plasmid, the translational state of the terminator changes from a translated state to a nontranslated state. Subsequently, the terminators change from a repressed state to an active state. Therefore, the methods described above do not reflect the termination effects under normal physiological conditions.

The second method, *in situ* detection of Rho-dependent terminators on chromosomes, is an approach to avoid the problem of translational state changes. Previously, researchers treated bacterial strains with the antibiotic bicyclomycin (BCM) to impair Rho activity (20), thereby increasing transcriptional read-through at RDT loci. Peters et al. examined changes in E. coli RNAP distribution in response to BCM by chromatin immunoprecipitation and microarray (ChIP-chip) as well as RNA sequencing (21, 22). Sequences in the read-through regions of transcription are considered to contain RDT sites. Using this method, the authors identified more than 1,200 RDT loci genome-wide. Subsequently, Chhakchhuak and Sen measured the termination efficiency of 72 Rho-dependent terminators in E. coli by quantitative reverse transcription-PCR (RT-qPCR) analyses (14). In this approach, primers were designed downstream of a suspected RDT site and the RDT regions were verified by comparing the RNA expression levels of a wild-type strain and a strain with impaired Rho activity. Indeed, the 3′ end of Rho-terminated mRNA is rapidly digested by 3′-to-5′ exonuclease digestion after RDT due to the lack of secondary structure (23). As a result, these identified sites are hundreds or thousands of bases long, far exceeding the length of Rho-dependent terminators. Those read-through regions in Rho-impaired strain actually consist of three parts: (i) the exonuclease-processed sequence; (i) the C-rich region and RDT site, and (iii) the read-through sequence downstream of the RDT site. Therefore, neither of these two methods can pinpoint RDT sites precisely. Most importantly, Dar and Sorek attempted to reveal unprocessed RNA 3′ ends by reducing 3′-to-5′ exonuclease activity in E. coli (24). However, various exonucleases, such as polynucleotide phosphorylase (PNPase), RNase II, and RNase R, could process the 3′ end of RNA (25, 26). Since they did not have access to double- or triple-deletion mutants (24, 25), they could not address the issue of exonuclease digestion.

Small RNAs (sRNAs) play an important role in regulating gene expression in response to extracellular stress by base-pairing with mRNA or other sRNAs (27–30). Most sRNAs are divided into two parts: a 3′ Hfq-binding scaffold sequence and a 5′ target-binding sequence (27, 31, 32). These structural motifs facilitate the development of synthetic sRNAs (sysRNAs) for gene regulation. For example, Na et al. designed a sysRNA with an 81-bp Hfq-binding MicC scaffold sequence in the 3′ portion and a 24-bp target-binding sequence in the 5′ portion (33). These sysRNAs were designed to repress target gene expression at the translational level (33–35). We note that sysRNA differs from natural sRNA in that the sequence at the 5′ end of sysRNA is fully complementary to the target mRNA, whereas natural sRNA is only partially complementary. Since exonuclease activity is inhibited by double-stranded RNA (dsRNA) (36), we hoped to stabilize the 3′ end of mRNA by inhibiting exonucleases activity through the sysRNA-mRNA interaction. We hypothesized that if the RNA 3′ end could be stabilized, RDT-terminated transcripts would no longer be degraded by exonucleases, which would greatly improve the accuracy of *in vivo* detection of RDT site.

In this study, to identify the *galM* RDT region *in vivo*, we designed a sysRNA with an approximately 20-bp target-binding sequence that is perfectly base paired with *galM* mRNA. Our results demonstrated that dsRNA formed by sysRNA-mRNA hybridization recruited the endonuclease RNase III to form a new mRNA 3′ end in the middle of the dsRNA region, thereby protecting the cleaved upstream mRNA fragment from rapid 3′-to-5′ exonuclease degradation and thus stabilizing the 3′ end of mRNA. We found that the intensity of the signal at the 3′ end of the RNA was proportional to the amount of mRNA expressed. Based on the above results, we designed and constructed four sysRNAs that bind to different regions upstream and downstream of the predicted *galM* RDT region. By measuring the signal intensities of these four newly generated 3′ ends, we verified a region of increased mRNA expression in the Rho-impaired strain, which was identified as the *galM* RDT region located within this 21-bp region. To our knowledge, this is the most accurate localization of the RDT region *in vivo*. We believe that this approach could be extended to identify other Rho-dependent terminators and is important for further understanding of RDTs.

## RESULTS

### Construction and overall design principles of sysRNA.

**FIG 1:**
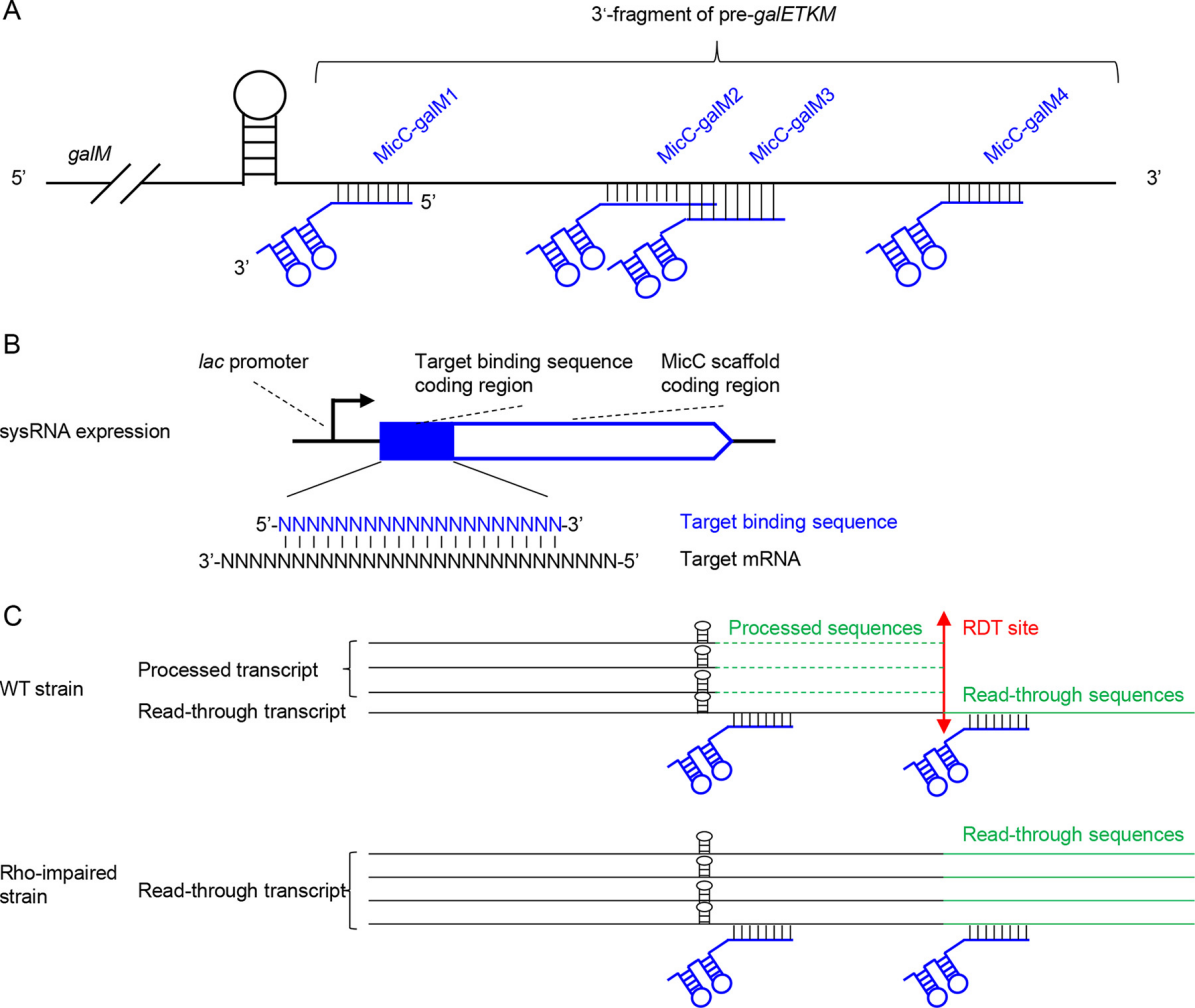
Schematic representation of overall design principles and sysRNA construction. (A) Schematic representation of the binding of four sysRNAs to pre-*galETKM* mRNA. (B) sysRNA was expressed under the control of the *lac* promoter. The coding region of the target binding sequence was inserted upstream of the coding region of the MicC scaffold. (C) In wild-type strains, RDT produces abundant terminated transcripts that are further processed by exonuclease to generate a stable 3′ end in the stem-loop structure, whereas the number of read-through transcripts is increased in Rho-impaired strains. Green dotted lines represent sequences that have been digested by exonucleases. Green solid lines represent read-through sequences.

To develop a method for locating RDTs *in vivo*, we used the Rho-dependent terminator downstream of *galM* as an example. In a previous study, we reported the presence of two tandem terminators, a Rho-independent terminator and a Rho-dependent terminator, located at +7 to +30 and +31 to +108 downstream of *galM*, respectively (the first base downstream of the *galM* translation termination site was set as +1) (23). Based on *in vitro* transcription results, RNAP undergoes a transcriptional pause at +111 and transcription termination at +124 (23). *In vivo*, we could not detect the exact *galM* RDT site but only a processed and relatively stable 3′-end signal at +28, close to the RIT stem-loop structure (23). To determine the exact RDT site *in vivo*, we constructed four MicC scaffold sysRNAs: MicC-galM1, MicC-galM2, MicC-galM3, and MicC-galM4. These four sysRNAs bound to distinct regions in the predicted Rho-dependent terminator downstream of the *galM* open reading frame (ORF), a 3′ fragment of the pre-*galETKM* (Fig. 1A). Further, the MicC scaffold sRNAs were expressed under the control of the *lac* promoter in the pBR322-derived plasmids (Fig. 1B). If the sysRNAs synthesized *in vivo* bound to the 3′ fragment of pre-*galETKM* mRNA, they would generate dsRNAs that prevent cleavage by exonucleases, resulting in new RNA 3′ end signals on the stabilized mRNA. Therefore, we expected that the binding of sysRNAs to the 3′ fragment of pre-*galETKM* mRNA would generate 3′ ends at specific residues at different locations. Notably, if the dsRNA regions blocked digestion of incoming 3′-to-5′ exonuclease, the detected 3′ ends of the mRNA should be a few nucleotides downstream of the sysRNA binding sites. We hypothesized that, in strains with impaired Rho activity, increased mRNA expression downstream of the RDT site would result in increased signals at the 3′ ends of mRNA. Therefore, comparing the signal intensity at the 3′ ends of wild-type and Rho-impaired strains could help us identify the RDT region *in vivo* (Fig. 1C).

### A new 3′-end signal was generated in the dsRNA region of the sysRNA-mRNA hybrid.

**FIG 2:**
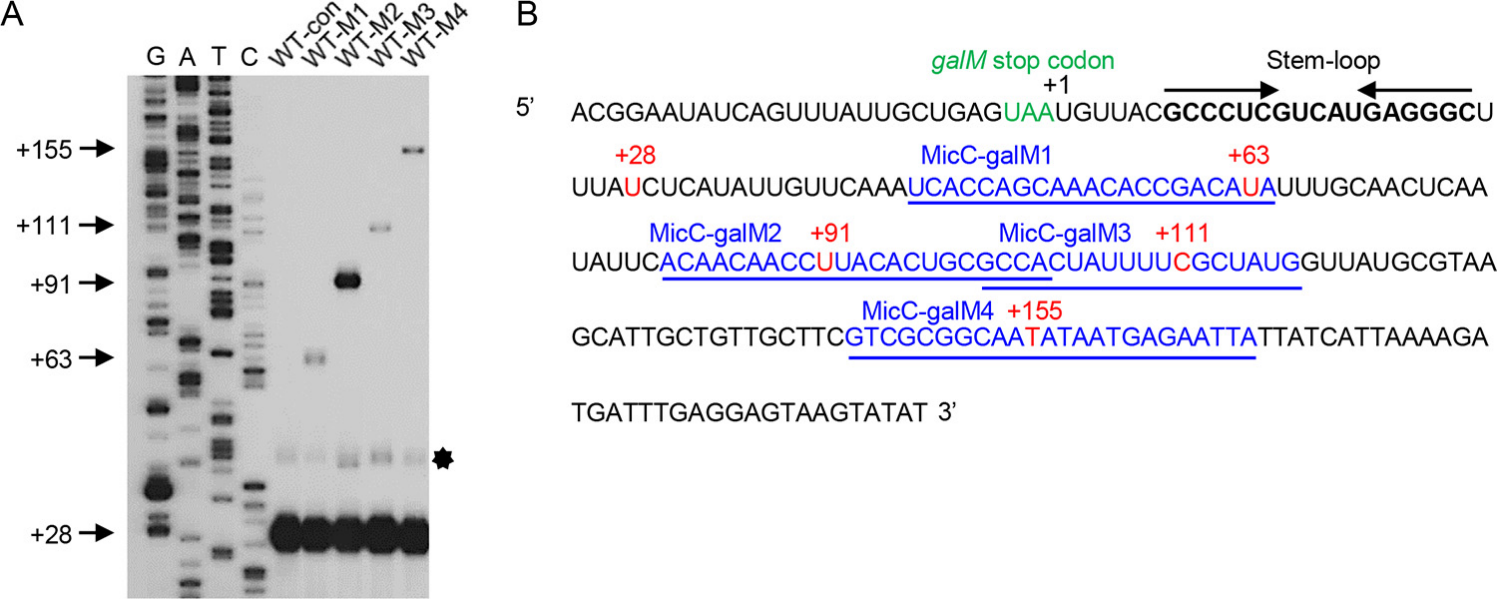
sysRNAs enabled visualization of the 3′ fragment of pre-*galETKM* and beyond. (A) 3′ RACE and primer extension assays of *gal* transcripts generated *in vivo* in WT-con and WT-Ms strains. Numbers on the left indicate the positions of the 3′ ends of the *gal* mRNA generated from sysRNA binding (the first base downstream of the *galM* translation termination site is set as +1). The DNA sequencing ladders in the four lanes are marked G, A, T and C. The bands indicated by the star are the +28 3′ end bands for which persistence of residual RNA secondary structures resulted in slower migration. (B) Sequence downstream of the *galM* stop codon. The mRNA sequences to which each sysRNA can base pair are indicated in blue and underlined. The 3′ ends of the RNA generated by synthetic sRNAs are shown in red. The *galM* stop codon is in green.

To determine whether sysRNAs can generate new 3′-end signals on the 3′ fragment of pre-*galETKM*, we extracted total RNA from wild-type E. coli MG1655 strains harboring MicC-galM expression plasmids (WT-Ms) and amplified mRNA 3′ ends using a rapid amplification of cDNA 3′ ends (3′ RACE) assay. Subsequently, we applied primer extension reactions using mixtures containing specific primers labeled with ^32^P to identify the sequences of interest in the amplified fragments. Finally, we performed capillary electrophoresis to visualize the fragments (37). In WT-con (control strain without sysRNA expression), no 3′ end was detected in the 3′ fragment of pre-*galETKM* mRNA (Fig. 2A), whereas a new 3′ end was generated in WT-M1, WT-M2, WT-M3, and WT-M4 strains (Fig. 2A). We also measured the length of DNA fragments by comparing the length of primer extension products to the length of the DNA sequencing ladders (Fig. 2A). Based on the length information, we mapped the 3′ ends of the mRNAs produced by sysRNA to the *gal* operon. The result showed that the sysRNAs MicC-galM1, MicC-galM2, MicC-galM3, and MicC-galM4 generated RNA 3′ ends at positions +63, +91, +111, and +155, respectively (Fig. 2A). By pinpointing the 3′ ends of mRNAs, we found that they were not downstream of the sysRNA binding sites. Instead, all 3′ ends were formed within the dsRNA region of the sysRNA-mRNA hybrids (Fig. 2B). These results demonstrated that, contrary to expectations, dsRNA did not help the generation of 3′ ends by blocking 3′-to-5′ exonuclease processing. Instead, it is endoribonuclease digestion that resulted in the formation of new 3′ ends, and the cleaved dsRNA further prevents the processing of exonucleases.

### 3′ ends were generated by RNase III cleavage.

**FIG 3:**
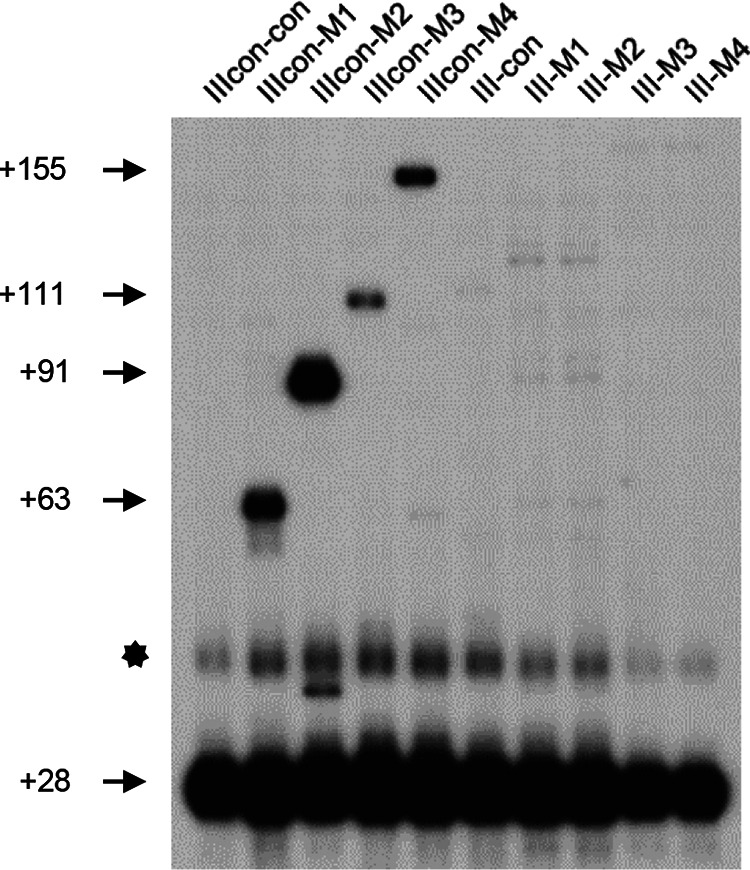
3′ RACE and primer extension assays of *gal* transcripts in IIIcon-con, IIIcon-Ms, III-con, and III-Ms strains. The numbers on the left indicate the positions of the 3′ ends of *gal* mRNA generated from sysRNA binding. The bands indicated by the star are the +28 3′ end bands for which persistence of residual RNA secondary structure resulted in slower migration.

To identify the endonuclease responsible for cleavage, we examined the RNA 3′ ends in the E. coli endonuclease mutant strains harboring various MicC-galMs plasmids: the RNase G *rng*::*cat* mutant (G-M1, G-M2, G-M3, and G-M4), the temperature-sensitive RNase E *ams1*^Ts^ mutant (E-M1, E-M2, E-M3 and E-M4), the temperature-sensitive RNase P *rnpA49* mutant (P-M1, P-M2, P-M3 and P-M4), and the RNase III *rnc* mutant (III-M1, III-M2, III-M3 and III-M4) (see Table S1 in the supplemental material) (31, 38). Although no 3′ end signal changes were observed in the G-Ms, E-Ms, and P-Ms strains, we found a change in the 3′-end signals between the IIIcon-Ms and III-Ms strains. In RNase III control strains harboring MicC-galM plasmids (IIIcon-M1, IIIcon-M2, IIIcon-M3, and IIIcon-M4), all four sysRNAs generated 3′ ends (Fig. 3), whereas in RNase III mutant strains harboring MicC-galM plasmids (III-M1, III-M2, III-M3, and III-M4), none of the sysRNAs generated 3′ ends (Fig. 3). Meanwhile, the only +28 3′ end of mature *galETKM* mRNA was detected from strains without sysRNA (IIIcon-con and III-con) (Fig. 3). These results indicated that the +63, +91, +111, and +155 3′ ends were generated by RNase III cleavage of the dsRNA between the sysRNA and the *gal* mRNA.

### Quantities of mRNA and Hfq affected 3′-end signal intensity.

**FIG 4:**
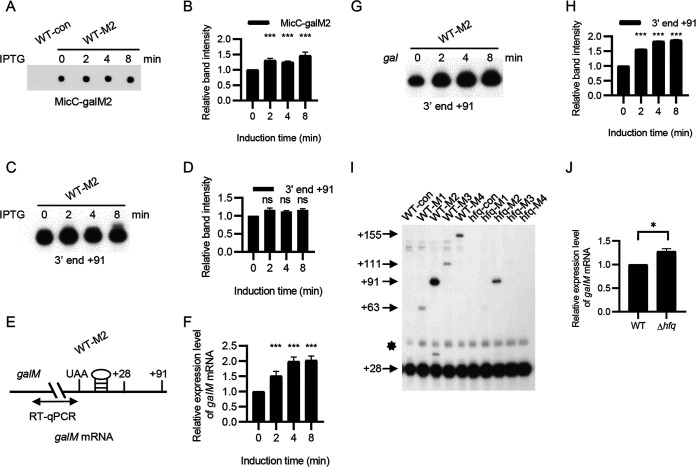
Factors affecting the 3′ end generation. (A) Expression of MicC-galM2 in WT-M2 strain at 0, 2, 4, and 8 min after IPTG induction. (B) Quantification of signal intensity of each band using ImageJ. The relative expression of MicC-galM2 after induction was normalized with the internal control, 16S rRNA, and is presented as a histogram. (C) Expression of the +91 3′ end in WT-M2 strain at 0, 2, 4, and 8 min after IPTG induction. (D) Quantification of signal intensity of each band using ImageJ. The relative expression of the +91 3′ end after induction is presented as a histogram. (E) Graph showing *galM* mRNA with primers (double arrow) amplifying the region upstream of the *galM* translation stop codon. The RNA stem-loop structure indicates a Rho-independent terminator. (F) The relative expression level of *galM* in the WT-M2 strain grown in LB after galactose induction was measured by RT-qPCR. (G) Expression of the +91 3′ end in the WT-M2 strain at 0, 2, 4, and 8 min after galactose induction. (H) The signal intensity of each band was quantified using ImageJ. The relative expression of the +91 3′ end after induction is presented as a histogram. (I) 3′ RACE and primer extension assays of *gal* mRNAs in the WT-con, WT-Ms, hfq-con, and hfq-Ms strains. The numbers on the left represent the positions of the 3′ ends. The bands indicated by the star are the +28 3′ end bands for which the persistence of residual RNA secondary structure resulted in slower migration. (J) Relative expression levels of *galM* in WT and Δ*hfq* strains grown in LB after galactose induction were measured by RT-qPCR. Data are means and standard deviations (SD) for 3 biological replicates. ns, not significant (*P* > 0.05); *, 0.01 < *P* < 0.05; ***, *P* < 0.001.

To investigate factors that influence signal intensity at the 3′ end of mRNA, we examined the following three factors: sysRNA expression level, mRNA expression level, and the ability of mRNA to bind sysRNA. We chose the +91 3′ end with the strongest 3′ end signal among the four sysRNAs as a representative to study the above three factors. First, we examined the effect of the amount of MicC-galM2 on the intensity of the +91 3′ end by culturing the WT-M2 strain in LB medium and inducing the expression of MicC-galM2 with isopropyl β-d-1-thiogalactopyranoside (IPTG). We then detected the expression of MicC-galM2 by dot blot assay. The results showed that there was no MicC-galM2 expression in the WT-con strain, while the expression of MicC-galM2 increased over time in the WT-M2 strain (Fig. 4A). Eight minutes after IPTG induction, it was 1.5 times that before induction (Fig. 4B). Notably, MicC-galM2 exhibited relatively high expression even before induction, likely due to transcriptional leakage. On the other hand, the expression of the +91 3′ end at 0 min was the same as that at 8 min after IPTG induction in the WT-M2 strain (Fig. 4C and D), suggesting that MicC-galM2 did not affect the expression of +91 3′ end when MicC-galM2 exceeded a certain threshold.

Second, we investigated the effect of the amount of *gal* mRNA on the intensity of the +91 3′ end. We quantified *galM* mRNA in the WT-M2 strain using RT-qPCR with primers designed to amplify a region of 100 bp upstream of the *galM* stop codon UAA (Fig. 4E). The results showed a 2-fold increase in *galM* mRNA expression after 4 and 8 min of induction with galactose (Fig. 4F). Meanwhile, the expression of +91 3′ end also gradually increased over time in the WT-M2 strain (Fig. 4G), and at 8 min, it was 2.0-fold higher than before induction (Fig. 4H). These results indicated that *gal* mRNA expression was positively correlated with that of the +91 3′ end.

Finally, we explored the effect of Hfq on the generation of +63, +91, +111, and +155 3′ ends. Hfq is a ubiquitous Sm-like RNA-binding protein that facilitates sRNA-mRNA interactions (39, 40). We compared the 3′ ends of WT-Ms strains with those of hfq-Ms strains (WT-M1, WT-M2, WT-M3 WT-M4, hfq-M1, hfq-M2, hfq-M3, and hfq-M4). There was little change in the expression of the +28 3′ end, whereas the signal intensity of the sysRNA-generated 3′ ends was lower in the hfq-Ms strains than in the WT-Ms strains (Fig. 4I). We also examined the expression of *galM* mRNA by RT-qPCR and found that the expression of *galM* mRNA in the Δ*hfq* strain was 1.3 times that of the WT strain, so the decrease of the 3′ ends signal was not caused by the reduced expression of *galM* mRNA in Δ*hfq* (Fig. 4J). These results suggested an important role for Hfq in sysRNA function.

The above results showed that the signal intensity at the 3′ end did not increase when sysRNA was overexpressed. However, when the amount of mRNA increased, the signal intensity at the 3′ end increased, and the expression of Hfq had a strong effect on the signal intensity at the 3′ end.

### Identification of the *galM* RDT locus *in vivo*.

**FIG 5:**
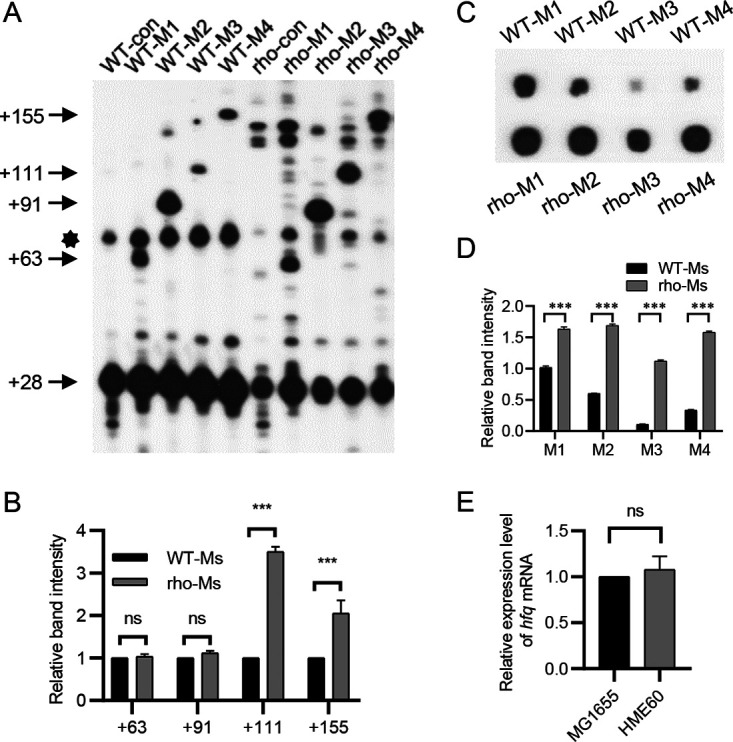
Determination of the *galM* RDT region using WT-Ms and rho-Ms strains. (A) 3′ RACE and primer extension assays of *gal* transcripts in the WT-con, WT-Ms, rho-con, and rho-Ms strains. The bands indicated by the star are the +28 bands for which the persistence of residual RNA secondary structure resulted in slower migration. (B) Quantification of signal intensity of the 3′ ends at positions +63, +91, +111, and +155 in panel A using ImageJ. The relative density of each band is presented as a histogram. (C) Expression of four sysRNAs in the WT-Ms and rho-Ms strains. (D) Quantification of signal intensity of each band using ImageJ. Relative band intensities were normalized to that of the internal control, 16S rRNA, and are presented in histograms. (E) Relative expression levels of *hfq* mRNA measured by RT-qPCR in strains MG1655 and HME60. Data are means and SD for 3 biological replicates. ns, not significant (*P* > 0.05); ***, *P* < 0.001.

To identify the RDT region *in vivo*, we chose to compare the signal intensity at the 3′ ends of positions +63, +91, +111, and +155 in the WT-Ms strains with those of the HME60-derivative rho-Ms strains with impaired Rho function (23, 41). We found that the signal intensity at the +63 3′ end was identical in the WT-M1 and rho-M1 strains, as was the +91 3′ end. However, the +111 and +155 3′ ends were increased 3.5-fold and 2.0-fold in rho-M3 and rho-M4 strains, respectively, compared to WT-M3 and WT-M4 strains (Fig. 5A and B). Meanwhile, the expression of the +28 3′ end was reduced in the rho-Ms strain. Next, we quantified the amount of sysRNA by dot blotting and found that the expression of four sysRNAs was significantly elevated in rho-Ms relative to WT-Ms strains (Fig. 5C and D). Although the expression of sysRNAs was significantly increased in rho-Ms, the expression at the +63 and +91 3′ ends was not, confirming our previous conclusion that the increase of sysRNA did not affect the signal intensity of mRNA 3′ end when there are too many sysRNAs. Likewise, we hypothesized that the increase in the +111 and +155 3′ end signals was not due to the increased expression of sysRNAs. We also examined the expression of *hfq* in MG1655 and HME60 strains by RT-qPCR, and the results showed that there was no significant change in the expression of *hfq* (Fig. 5E). From the above findings, we deduced that the increases in signals at the +111 and +155 3′ ends were due to increased mRNA expression. Therefore, the RDT loci *in vivo* could be located in the region between positions +91 and +111.

**FIG 6:**
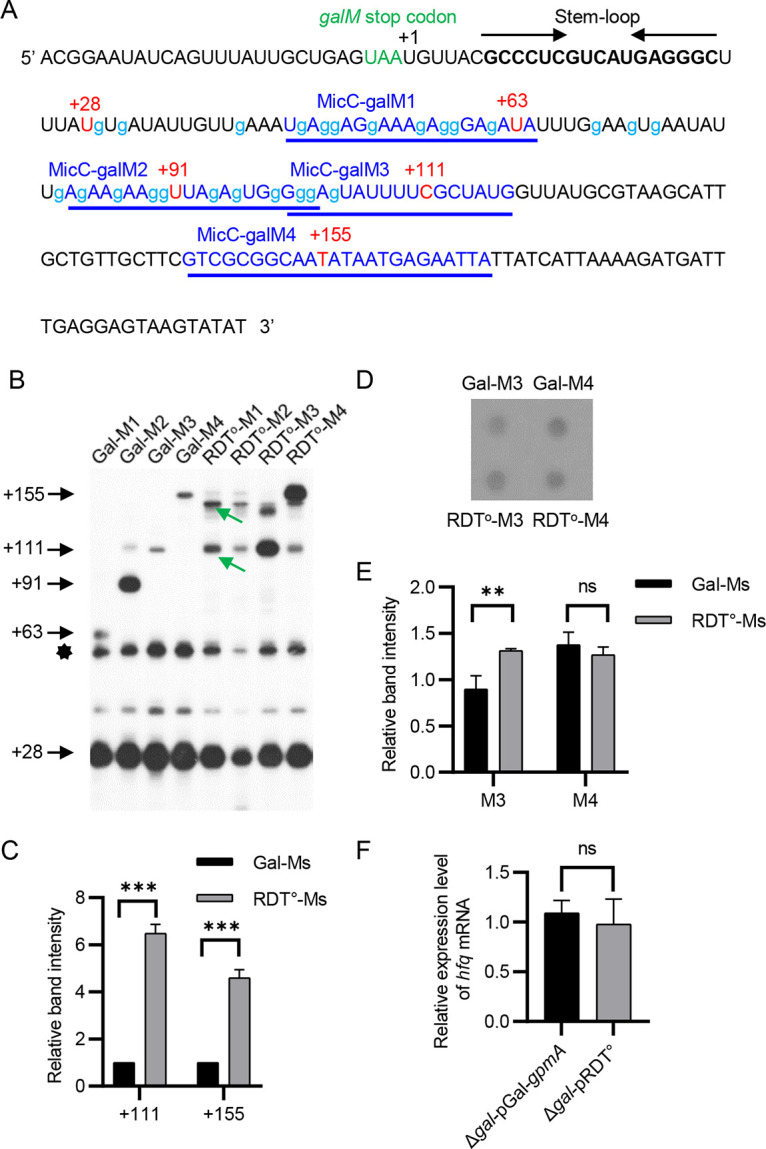
Determination of the *galM* RDT region using Gal-Ms and RDT^o^-Ms strains. (A) Sequence downstream of the *galM* stop codon in the pRDT^o^ plasmid. Lowercase “g” represents mutated cytosines. Those cytosines in the pGal plasmid were mutated to guanines. (B) 3′ RACE and primer extension assays of *gal* transcripts in the Gal-Ms and RDT^o^-Ms strains. The bands indicated by the star are the +28 bands for which the persistence of residual RNA secondary structure resulted in slower migration. Green arrows indicate new 3′ ends that may arise from changes in secondary structure due to changes in the RNA sequence. (C) The signal intensities of +111 and +155 3′ ends in panel B were quantified using ImageJ. The relative density of each band is presented as a histogram. (D) Expression of MicC-galM3 and MicC-galM4 in Gal-M3, Gal-M4, RDT^o^-M3 and RDT^o^-M4 strains. (E) The signal intensity of each dot in panel D was quantified using ImageJ. Relative band intensities were normalized with the internal control, 16S rRNA, and are presented in a histogram. (F) Relative expression levels of *hfq* mRNA measured in the Δ*gal*-pGal and Δ*gal*-pRDT^o^ strains. Data are means and SD for 3 biological replicates. ns, not significant (*P* > 0.05); **, 0.001 < *P* < 0.01; ***, *P* < 0.001.

To further confirm our conclusions, we revalidated the RDT loci of mutant strains in the C-rich region. We first cloned the coding region from the galactose operon to *gpmA* on the MG1655 genome into the single-copy plasmid pCC1BAC to generate the pGal-*gpmA* plasmid. We then replaced all cytosines in the C-rich region of the *galM* Rho-dependent terminator with guanines (Fig. 6A) to generate the pRDT^o^ plasmid (23). Finally, these plasmids were individually introduced into the galactose operon deletion (Δ*gal*) strain for subsequent testing. We named these two derivative strains Δ*gal*-pGal-*gpmA* and Δ*gal*-pRDT^o^, respectively. Δ*gal*-pRDT^o^ has been reported to be incapable of *galM* RDT (23). The plasmid pMicC-galM was transformed into Δ*gal*-pGal-*gpmA* and Δ*gal*-pRDT^o^ for subsequent analysis, and the resulting strains were named Gal-Ms and RDT^o^-Ms, respectively. Due to mutation in *gal* mRNA, MicC-galM1 and MicC-galM2 were not fully complementary to the target mRNA, and we could not observe the +63 and +91 3′ ends in the RDT^o^-M1 and RDT^o^-M2 strains (Fig. 6B). Meanwhile, we observed 6.5- and 4.6-fold increases in the +111 and +155 3′ ends of the RDT^o^-M3 and RDT^o^-M4 strains compared to the Gal-M3 and Gal-M4 strains, respectively (Fig. 6B and C). Dot blot assays of MicC-galM3 and MicC-galM4 showed that while the expression of MicC-galM3 was slightly increased in the RDT^o^-M3 strain, the expression of MicC-galM4 in the RDT^o^-M4 strain was not significantly changed compared to Gal-M3 and Gal-M4 strains (Fig. 6D and E). Finally, we examined the expression of *hfq* in Δ*gal*-pGal-*gpmA* and Δ*gal*-pRDT^o^ strains by RT-qPCR and found no significant change in the expression of *hfq* in the two strains (Fig. 6F). Taken together, our observations led us to conclude that the *galM* RDT site was located in the region between positions +91 and +111.

### Targeting sites selection for sysRNA.

As shown by our results, each MicC-galM construct generated a different number of RNA 3′ ends. The number of +91 3′ ends was much higher than that of +63 3′ ends (Fig. 2). However, dot blot quantification of each MicC-galM construct showed no correspondence between the amount of MicC-galM RNA and the 3′ end (Fig. 5C). To clarify the reasons for the difference in 3′-end generation, we analyzed the possible reason from the perspective of *gal* mRNA. First, the two MicC-galM1 and MicC-galM2 targeting sites were located within the C-rich region of the *galM* Rho-dependent terminator upstream of the RDT site. Therefore, we believe that the two sysRNAs would bind the same amount of *gal* mRNA in this narrow region.

**FIG 7:**
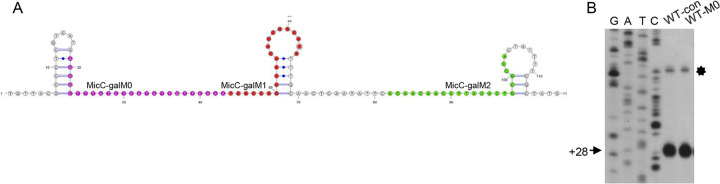
mRNA secondary-structure analysis and 3′ end visualization. (A) Secondary-structure prediction of *galM* mRNA extending from +1 to +117 at the 3′ end. Strong stem-loop structure in the Rho-independent terminator of *galM* and two weak stem-loop structures in the C-rich region. Target-binding sequence for MicC-galM0 (purple), MicC-galM1 (red), and MicC-galM2 (green). (B) 3′ RACE and primer extension assays for *gal* transcripts in the WT-con and WT-M0 strains. The DNA sequencing ladders in the four lanes are labeled G, A, T, and C. The bands indicated by the star are the +28 bands for which the persistence of residual RNA secondary structure resulted in slower migration.

Next, we analyzed the secondary structure of *gal* mRNA. Using the RNAfold web server (42), we investigated the secondary structure of this mRNA extending from +1 to +117 at the 3′ end (Fig. 7A). The RNAfold web server predicted the formation of a 5-bp stem with a 10-bp loop and a 3-bp stem with a 10-bp loop at positions +50 to +69 and +98 to +113, respectively. Furthermore, no other secondary structure was observed within the mRNA segment. The minimum free energies (MFE) of these two stem-loops were −0.90 kcal/mol (5-bp stem with 10-bp loop) and −1.00 kcal/mol (3-bp stem with 10-bp loop). The target-binding regions of MicC-galM1 and MicC-galM2 in the fragment showed 15 (of 21) and 6 (of 22) base pair sequences in these two secondary structures, respectively (Fig. 7A). Accordingly, approximately 71% of MicC-galM1-targeted mRNA sequences and 27% of MicC-galM2-targeted mRNA sequences were located in the RNA secondary structures, suggesting that MicC-galM2 could bind target mRNAs better than MicC-galM1.

The relative cleavage efficiency of dsRNA by RNase III was in good agreement with the percentage of base pairing in the RNA secondary structures; specifically, the RNase III cleavage efficiency on dsRNA generated by MicC-galM2 was higher than that of MicC-galM1. To further reveal the effect of secondary structure on cleavage efficiency, we selected the *galM* RIT region containing a robust secondary structure with an MFE of −10.30 kcal/mol as the target of the new sysRNA MicC-galM0. As expected, MicC-galM0 failed to produce a new 3′ end in the WT-M0 strain (Fig. 7B). This could be explained by the inhibition of sysRNA function by secondary structures on target mRNA. Therefore, the secondary structure of the target mRNA should be considered when designing the target binding sequence to ensure that the sysRNA binds the target mRNA in regions free of secondary structure.

## DISCUSSION

In this study, we demonstrated that dsRNA formed by sysRNA binding to mRNA recruited RNase III for dsRNA cleavage and that the cleaved RNA 3′ end is resistant to further exonuclease digestion, thereby stabilizing the RNA 3′ end that was rapidly degraded in the absence of sysRNA. By constructing four sysRNAs that bind to different sites of mRNA, we observed four distinct stable RNA 3′ ends. When the RNA 3′ ends were located upstream of the RDT site, the signal intensity at the 3′ ends of WT-Ms generated by sysRNA was unchanged compared to that of the rho-Ms strains. However, when the RNA 3′ ends were located downstream of the RDT site, the signal intensity at the RNA 3′ ends increased due to the increased expression of downstream mRNA in the rho-Ms strains. With this approach, we identified *in vivo* the RDT region of the last gene of the galactose operon *galM*.

### Implementation of a larger-scale sysRNA-based RDT region detection strategy.

Tandem terminations where both RITs and RDTs are involved in the termination process are attracting attention (43, 44). The RDTs in these tandem terminators can be further classified as pure RDTs and hybrid RDTs. For pure RDTs, Rho does not directly stimulate RIT; however, processing of RDT-terminated transcripts downstream of RIT will result in altered RIT termination efficiency. This phenomenon leads to an indirect stimulatory effect of Rho on RIT. For hybrid RDTs, Rho stimulates termination at Rho-independent terminators by remodeling the RNA upstream of the Rho-independent terminators to prevent the formation of RNA structures that could otherwise compete with the terminator hairpin (43). In these cases, Rho showed a direct activating effect on RIT.

We asked whether a sysRNA-based strategy could be applied to distinguish pure RDT from hybrid RDT. From the study described above, we can speculate that the most difficult aspect of distinguishing pure RDT from hybrid RDT is the exclusion of exonuclease interference. Given our results showing that RNase III-cleaved dsRNA could inhibit exonuclease processing of RNA 3′ ends, we hypothesized that it would be possible to distinguish them by comparing the RIT efficiency in sysRNA-expressing WT and Δ*rho* derivative strains. If the RIT efficiency of the sysRNA-expressing Δ*rho* strain is lower than that of the sysRNA-expressing WT strain, then the RDT should be a hybrid RDT; otherwise, the RDT should be a pure RDT. The *galM* terminator used in this study is one of the tandem terminators in E. coli. We did observe a decrease in the +28 RIT 3′ end of the rho-Ms strains compared to the WT-Ms strains (Fig. 5A). Therefore, we speculated that *galM* RDT is a hybrid RDT.

In E. coli, approximately 20 to 30% of genes are terminated by Rho (21, 24). Among these Rho-dependent terminators are those located at the ends of genes (intergenic) and those located within genes (intragenic) (21). The *galM* Rho-dependent terminator proposed in this study can be used as a proxy for intergenic terminator for testing of sysRNA-based strategies. However, it is unclear how well the sysRNA-based strategy works for intragenic Rho-dependent terminator detection. To answer this question, we constructed several sysRNAs with targeting sites located at the end of *galE* mRNA and the beginning of *galT* mRNA, upstream and downstream of the predicted RDT region within the *galT* gene (45). The results showed that none of the sysRNAs produced 3′ ends. We attributed this to more extensive translation events in intragenic regions than in intergenic regions (46–48). Translating ribosomes temporarily stalled at sysRNA target sites may hinder sysRNA access to mRNA, thereby preventing RNase III cleavage to form 3′ ends. Therefore, the occupancy of target regions on mRNA by translating ribosomes should be considered in practical applications. Furthermore, as mentioned above, the local secondary structure of mRNA may prevent the binding of sysRNA to mRNA, rendering this detecting system ineffective. Therefore, it is necessary to consider the above two points when this system is used for the detection of specific RDT site.

### Possible reasons for the inconsistency of RDT loci *in vitro* and *in vivo*.

Our previous results showed that the *galM* RDT site is located at +124 *in vitro* (23); however, our current sysRNA-based strategy suggested that the *galM* RDT region was located between +91 and +111 *in vivo*. We tried to determine why these results were inconsistent. It has been reported that the transcription process is highly dependent on a series of *in vivo* cofactors (49). Several studies of bacterial transcription elongation reveal that transcription rates are synchronized with translation rates (50–52). Therefore, a lack of translation *in vitro* might result in transcription rates different from those *in vivo*. Moreover, the binding of the N-terminal domain of NusG to RNAP might prevent the polymerase from entering long pauses, thereby increasing the overall transcription rate (53). Our unpublished data suggested that NusG might promote *galM* RDT. In addition to this, *in vivo* factors, such as NusA and Gre proteins, known to regulate pausing, stalling, and termination can regulate the RNAP elongation and termination process (54–56). Since RNAP pausing is a crucial step in the RDT process, changes in transcription rates can lead to differences in pausing sites, which may result in differences in RDT sites. Therefore, we hypothesized that changes in these factors in the *in vivo* and *in vitro* environments could lead to differences in RDTs. Indeed, due to the inability to pinpoint transcription termination sites *in vivo*, no case was reported comparing *in vivo* and *in vitro* RDT sites. It is for these reasons that it is important to develop a method that can detect RDTs *in vivo* without altering their physiological environment. Of these, the current sysRNA-based strategy has the following advantages: (i) high specificity, (ii) ease of design and implementation, and (iii) minimal impact on cell growth. Therefore, our method might open up space for further investigations of RDTs *in vivo*.

### Further expansion of sysRNA applications.

Natural sRNAs serve a variety of physiological purposes, but the use of sysRNAs is still limited to inhibiting gene expression. Here, we used sysRNAs for the first time to detect RDT loci, extending their applications. In eukaryotes, RNA editing is an area of great interest. Compared to DNA editing, RNA editing does not require permanent changes to the genome, and this reversible and easily regulated editing method may have advantages in terms of safety. A major problem with editing technologies that rely on a CRISPR/Cas system is their dependence on the expression of exogenous editing enzymes or effector proteins. This can lead to issues such as cytotoxicity or delivery difficulties (57, 58). Wei’s research group discovered for the first time that specially designed RNAs have great potential for RNA editing and can edit target gene transcripts by recruiting endogenous RNA adenosine deaminase to produce efficient and precise editing of specific adenosines without introducing any exogenous effector proteins (59, 60). According to our results, sysRNA could cleave target mRNA by recruiting endogenous RNase III. We believe that the diverse functions of sysRNA-based strategies, such as RNA editing in bacteria, could also be achieved through appropriate modification of RNase III.

## MATERIALS AND METHODS

### Bacterial strains and growth conditions.

E. coli strains MG1655 and HME60 and the Δ*gal* and Δ*hfq* mutants (23, 38) (Table S1) containing the corresponding plasmids (indicated in the figures) were grown at 37°C in LB medium (containing 10 g tryptone, 5 g yeast extract, and 10 g NaCl per L of water) supplemented with 0.5% (wt/vol) galactose, chloramphenicol (15 μg/mL), ampicillin (100 μg/mL), or IPTG (1 mM) as necessary. Due to the leaky expression of MicC-galMs, we did not add IPTG to the medium except for IPTG induction experiments. Endonuclease-deficient strains and their parent strains, including SDF204 (*rnc^+^*; RNase III control), SDF205 (*rnc*; ΔRNase III), GW10 (*rng^+^ rne*^+^; control for RNase G and RNase E), GW11 (*rng*::*cat*; ΔRNase G), GW20 (*ams1*^Ts^; RNase E temperature-sensitive mutant), NHY312 (*rnpA^+^*; control for RNase P), and NHY322 (*rnpA49*; RNase P temperature-sensitive mutant) were generous gifts from Y. H. Lee (KAIST, South Korea) (31, 38) (Table S1). These strains containing the corresponding plasmids were grown at appropriate temperatures (30°C, 37°C, and 44°C) in LB medium supplemented with ampicillin (100 μg/mL).

### Plasmid construction.

The pGal plasmid is a derivative of pCC1BAC (Epicenter Biotechnologies, USA) containing a coding region from *galETKM* to *gpmA* for *gal* mRNA expression. On the pRDT^o^ plasmid, all cytosines in the *galM* RDT are replaced by guanines (23). To construct sysRNA expression plasmids (pMicC-galM1, pMicC-galM2, pMicC-galM3, and pMicC-galM4), the 81-bp MicC scaffold DNA fragment of MicC was first amplified by PCR using E. coli genomic DNA as a template, and then the PCR products were digested with EcoRI and HindIII and inserted into pLac (pBR322-derived plasmid) (61) to obtain the pHL1722 plasmid. Following that, 20-bp sequences complementary to the *gal* operon regions from positions +44 to +64, +82 to +102, +100 to +117, and +145 to +168 were inserted between the AatII and EcoRI sites of the pHL1722 plasmid (Fig. S1) to generate sysRNA expression plasmids. Plasmids used are listed in Table S2.

### Total RNA preparation and 3′ RACE assay.

Total RNA was extracted from 2 mL of E. coli strains as previously described (41). For the 3′ RACE assay, total RNA (10 μg) was ligated with 2 nM synthetic RNA oligomers (Table S3) with 5′ phosphate and 3′ inverted deoxythymidine (Integrated DNA Technologies, USA). RNA ligation was performed using 10 U of T4 RNA ligase (Thermo Fisher Scientific, USA) in a volume of 20 μL for 3 h at 37°C, supplemented with 20 U of RNasin RNase inhibitor (Promega, USA) to prevent RNase activity. The ligated RNA was purified using a G-50 column (GE Healthcare, USA), and 1 μg of RNA was reverse transcribed in a 20-μL reaction volume at 37°C for 2 h. The reverse transcription reaction mixture contained 4 U of Omniscript reverse transcriptase (Qiagen, Germany), a 0.5 mM concentration of a deoxynucleoside triphosphate (dNTP) mixture, a 0.4 μM concentration of the 3RP primer, which is complementary to the RNA oligomer (Table S3), and 10 U of RNasin. Finally, a 2-μL sample of this reaction mixture was used as the template for PCR. A 150-bp galactose operon-specific fragment containing the predicted region of the RNA 3′ end was amplified using HotStarTaq DNA polymerase (Qiagen, Germany) with 3RP primer and the *gal*-specific primer M3-F (Table S3).

To visualize the mRNA 3′ ends, the PCR products were purified and used as templates for primer extension reactions, which were performed in a volume of 20 μL using 1 U of *Taq* polymerase (Qiagen, Germany), a 0.2 mM concentration of a dNTP mixture, 0.3 μL of the ^32^P-labeled *gal*-specific primer M4-F (Table S3), and 2 μL of PCR product. Reaction products were separated on 8% polyacrylamide/urea sequencing gel. Radioactive bands were visualized by exposure of the gel to X-ray film, and the intensities of the radioactive signals were quantified using a PhosphorImager (Amersham Biosciences Corp., England). Data were subjected to one-way analysis of variance (ANOVA) using the Bonferroni test (*n* = 3). GraphPad Prism 9.0 was used for one-way ANOVA and graph plotting.

### RNA dot blotting.

For RNA dot blotting, total RNA was extracted, and 10 μg of this was treated with DNase I (TaKaRa, Japan) and purified with a G-50 column (GE Healthcare, USA). One microgram of RNA was then mixed with 2× RNA loading buffer and incubated at 80°C for 5 min to remove the RNA secondary structure and inactivate DNase I. RNA was spotted onto a positively charged nylon membrane (Thermo Fisher Scientific, USA). The membrane was adhered to Whatman filter paper and baked at 80°C for 1 h. The MicC probe (Table S3) was labeled with ^32^P, mixed with ULTRAhyb ultrasensitive hybridization buffer (Thermo Fisher Scientific, USA), and hybridized with RNA extract on a nylon membrane overnight at 42°C according to the manufacturer’s instructions (31, 38). Afterwards, the membrane was washed twice with 2× SSC (1× SSC is 0.15 M NaCl plus 0.015 M sodium citrate) containing 0.1% SDS at 25°C for 5 min and then washed twice with 0.2× SSC containing 0.1% SDS at 42°C for 15 min. Radioactive dots on the membrane were visualized by exposure to X-ray film. The signal intensity of the dots was quantified using the software ImageJ. 16S rRNA was selected as an internal reference gene and quantified by RT-qPCR. The relative expression levels of these dots were normalized to the internal control 16S rRNA. Data were subjected to one-way ANOVA using the Bonferroni test (*n* = 3). GraphPad Prism 9.0 was used for one-way ANOVA and graph plotting.

### RT-qPCR.

For RT-qPCR experiments, 2-mL samples from E. coli strains were collected. Total RNA was extracted, and RT-qPCR was conducted essentially as previously described (41). Results for each strain were normalized to that of the *rrsB* gene encoding 16S rRNA. For data analysis, technical and biological triplicate data were obtained. ΔΔ*C_T_* values were subjected to ANOVA using the Bonferroni test (*n* = 3). GraphPad Prism 9.0 was used for one-way ANOVA and graph plotting.
