# Supplementary material for: Identification of a Rho-Dependent Termination Site In Vivo Using Synthetic Small RNA
Source: Microbiol Spectr. 2023 Jan 18;11(1):e03950-22. doi: 10.1128/spectrum.03950-22 (PMC9927376; doi:10.1128/spectrum.03950-22)
Supplement: Supplemental file 1 — Supplemental material. Download spectrum.03950-22-s0001.pdf, PDF file, 0.3 MB [file spectrum.03950-22-s0001.pdf]

Supplementary files

For

Identification of Rho-dependent termination site *in vivo* using synthetic sRNA

Xun Wang[1*], Monford Paul Abishek N[2], Heung Jin Jeon[2,3], Jin He[1] and Heon M. Lim[2*]

[1]State Key Laboratory of Agricultural Microbiology, College of Life Science and Technology, Huazhong Agricultural University, Wuhan, Hubei 430070, [2]Department of Biological Sciences, College of Biological Sciences and Biotechnology, Chungnam National University, Daejeon 34134, Republic of Korea, PR China, [3]Infection Control Convergence Research Center, Chungnam National University College of Medicine, Daejeon 35015, Republic of Korea

Contents

1. Supplementary Figures S1

Supplementary Figure S1 Construction of synthetic sRNA (sysRNA) expressing plasmid…1

2. Supplementary Tables S1-S3

Supplementary Tables S1 Strains used in the study………..……...…...............…........…...2

Supplementary Tables S2 Plasmids used in this study…………………………………………4

Supplementary Tables S3 Primers used in this study……………………………………….....5

3. Supplementary References

Supplementary References…………………………………………….…………….………….6

Supplementary Figure S1

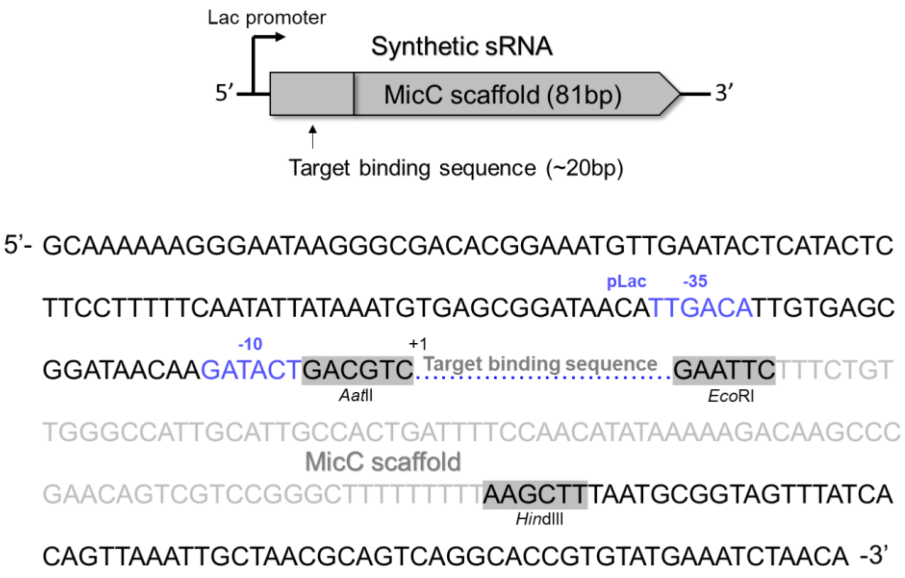

Supplementary Figures S1 Construction of synthetic sRNA (sysRNA) expressing plasmid

The sysRNA consists of two parts: a 20 bp target binding sequence at the 5'-end and an 81 bp MicC scaffold at the 3'-end. sysRNA was expressed under the control of the *lac* promotor. The -35 and -10 regions of the *lac* promoter are indicated in blue. The target-binding region coding sequence (light grey) is inserted between *Aat*II and *Eco*RI (blue dashed lines), and the MicC scaffold (indicated in gray) is inserted between *Eco*RI and *Hin*dIII. Recognition sites of restriction enzymes *Aat*II, *Eco*RI, and *Hin*dIII are highlighted in gray.

Supplementary Tables S1 Strains used in the study

Strain	Description	Source
MG1655	K-12 F⁻ λ⁻ *ilvG⁻ rfb-50 rph-1*	(1)
Δ*gal*	*galETKM* and *gpmA* gene deleted in the genome	(2)
Δ*hfq*	*hfq* gene deleted in the genome	(3)
HME60	W3110 derivative strain, K-12 F⁻ λ⁻ *rph*-1 INV (*rrnD*, *rrnE*) *rho*::*amp*^{r}	(1)
SDF204	*rnc+*; RNase III control	(4)
SDF205	*rnc-*; ΔRNase III	(4)
GW10	*rng+, rne+*; control for RNase G and RNase E	(4)
GW11	*rng::cat*; ΔRNase G	(4)
GW20	*ams1*^{ts}; RNase E temperature-sensitive mutant	(4)
NHY312	*rnpA+*; control for RNase P	(4)
NHY322	*rnpA49*; RNase P temperature-sensitive mutant	(4)
WT-con	MG1655 strain harboring pLac plasmid	This study
WT-M1	MG1655 strain harboring pMicC-galM1 plasmid	This study
WT-M2	MG1655 strain harboring pMicC-galM2 plasmid	This study
WT-M3	MG1655 strain harboring pMicC-galM3 plasmid	This study
WT-M4	MG1655 strain harboring pMicC-galM4 plasmid	This study
IIIcon-con	SDF204 strain harboring pLac plasmid	This study
IIIcon-M1	SDF204 strain harboring pMicC-galM1 plasmid	This study
IIIcon-M2	SDF204 strain harboring pMicC-galM2 plasmid	This study
IIIcon-M3	SDF204 strain harboring pMicC-galM3 plasmid	This study
IIIcon-M4	SDF204 strain harboring pMicC-galM4 plasmid	This study
III-con	SDF205 strain harboring pLac plasmid	This study
III-M1	SDF205 strain harboring pMicC-galM1 plasmid	This study
III-M2	SDF205 strain harboring pMicC-galM2 plasmid	This study
III-M3	SDF205 strain harboring pMicC-galM3 plasmid	This study
III-M4	SDF205 strain harboring pMicC-galM4 plasmid	This study
hfq-con	Δ*hfq* strain harboring pLac plasmid	This study
hfq-M1	Δ*hfq* strain harboring pMicC-galM1 plasmid	This study
hfq-M2	Δ*hfq* strain harboring pMicC-galM2 plasmid	This study
hfq-M3	Δ*hfq* strain harboring pMicC-galM3 plasmid	This study
hfq-M4	Δ*hfq* strain harboring pMicC-galM4 plasmid	This study
rho-con	HME60 strain harboring pLac plasmid	This study
rho-M1	HME60 strain harboring pMicC-galM1 plasmid	This study
rho-M2	HME60 strain harboring pMicC-galM2 plasmid	This study
rho-M3	HME60 strain harboring pMicC-galM3 plasmid	This study

rho-M4	HME60 strain harboring pMicC-galM4 plasmid	This study
Gal-M1	Δ*gal* strain harboring pGal-gpmA and pMicC-galM1 plasmids	This study
Gal-M2	Δ*gal* strain harboring pGal-gpmA and pMicC-galM2 plasmids	This study
Gal-M3	Δ*gal* strain harboring pGal-gpmA and pMicC-galM3 plasmids	This study
Gal-M4	Δ*gal* strain harboring pGal-gpmA and pMicC-galM4 plasmids	This study
RDT^{o}-M1	Δ*gal* strain harboring pRDT^{O} and pMicC-galM1 plasmids	This study
RDT^{o}-M2	Δ*gal* strain harboring pRDT^{O} and pMicC-galM2 plasmids	This study
RDT^{o}-M3	Δ*gal* strain harboring pRDT^{O} and pMicC-galM3 plasmids	This study
RDT^{o}-M4	Δ*gal* strain harboring pRDT^{O} and pMicC-galM4 plasmids	This study

Supplementary Tables S2 Plasmids used in the study

Plasmid	Description	Source
pLac	Plasmid pBR322 with *lac* promoter coding sequence inserted downstream of penicillin beta-lactamase coding gene, Amp^R	(3, 5, 6)
pHL1722	pLac plasmid containing MicC scaffold coding sequence inserted between *Eco*RI and *Hin*dIII, Amp^R	This study
pMicC-galM0	pHL1722 plasmid containing *galM* binding region coding sequence inserted between *Aat*II and *Eco*RI, Amp^R	This study
pMicC-galM1	pHL1722 plasmid containing *galM* binding region coding sequence inserted between *Aat*II and *Eco*RI, Amp^R	This study
pMicC-galM2	pHL1722 plasmid containing *galM* binding region coding sequence inserted between *Aat*II and *Eco*RI, Amp^R	This study
pMicC-galM3	pHL1722 plasmid containing *galM* binding region coding sequence inserted between *Aat*II and *Eco*RI, Amp^R	This study
pMicC-galM4	pHL1722 plasmid containing *galM* binding region coding sequence inserted between *Aat*II and *Eco*RI, Amp^R	This study
pGal-*gpmA*	The pCC1BAC plasmid contains the genes coding *galETKM* and *gpmA,* Cm^R	(2)
$pRDT^o$	The pCC1BAC plasmid contains the genes encoding *galETKM* and *gpmA*, and the cytosine in the *galM* RDT was replaced by guanine.	(2)

Supplementary Tables S3 Primers used in the study.

Primer name	Primer sequences (5′→3′)	Usage
Synthetic RNA oligomer	UUCACUGUUCUUAGCGGCCGCAUGCUC	Oligomer for RNA ligation used in 3' RACE
3RP	AGCATGCGGCCGCTAAGAAC	Reverse transcription and PCR primer for 3′ RACE assay
M3-F	TCCGCACGACGGCCTGAAAT	PCR primer for 3′ RACE assay
M4-F	CGAAGAGTATTCCAGCCTG	Primer extension assay
MicC-Probe	GGAAAATCAGTGGCAATGCAATGGCCCAAC	Dot blotting
MicC-F	GGGAATTCTTTCTGTTGGGCCATTG	pHL1722 construction
MicC-R	GGA AGC TTA AAA AAA AAG CCC GGA CG	
M-S0-F	CTTTGAACAATATGAGATAAAGCCCTG	pMicC-galM0 construction
M-S0-R	AATTCAGGGCTTTATCTCATATTGTTCAAAGACGT	
M-S1-F	CTATGTCGGTGTTTGCTGGTGAG	pMicC-galM1 construction
M-S1-R	AATTCTCACCAGCAAACACCGACATAGACGT	
M-S2-F	CGGCGCAGTGTAAGGTTGTTGTG	pMicC-galM2 construction
M-S2-R	AATTCACAACAACCTTACACTGCGCCGACGT	
M-S3-F	CCATAACCATAGCGAAAATAGTGGCG	pMicC-galM3 construction
M-S3-R	AATTCGCCACTATTTTCGCTATGGTTATGGACGT	
M-S4-F	CTAATTCTCATTATATTGCCGCGACG	pMicC-galM4 construction
M-S4-R	AATTCGTCGCGGCAATATAATGAGAATTAGACGT	
plac-R	TTAACTGTGATAAACTACC	Plasmid sequencing
galM-FP	TTTCTACCGGACAGCCCGAA	*galM* RT-qPCR
galM-RP	AAACTGA TA TTCCGTCAGGCTGGA	
hfq-FP	GCAAGGGCAAATCGAGTCTT	*hfq* RT-qPCR
hfq-RP	CGAGACGGGACAACAGTAGA	
rrsB-FP	TGGCGCATACAAAGAGAAGC	*rrsB* RT-qPCR
rrsB-RP	ACTCCAATCCGGACTACGAC	

Supplementary References

1. Lee HJ, Jeon HJ, Ji SC, Yun SH, Lim HM. 2008. Establishment of an mRNA gradient depends on the promoter: An investigation of polarity in gene expression. J Mol Biol 378:318-327.
2. Wang X, N MPA, Jeon HJ, Lee Y, He J, Adhya S, Lim HM. 2019. Processing generates 3' ends of RNA masking transcription termination events in prokaryotes. Proc Natl Acad Sci U S A 116:4440-4445.
3. Wang X, Ji SC, Lim HM. 2015. Two-level inhibition of *galK* expression by Spot 42: degradation of mRNA mK2 and enhanced transcription termination before the *galK* gene. Proc Natl Acad Sci U S A 112:7581-7586.
4. Wang X, Ji SC, Yun SH, Jeon HJ, Kim SW, Lim HM. 2014. Expression of each cistron in the *gal* operon can be regulated by transcription termination and generation of a *galK*-specific mRNA, mK2. J Bacteriol 196:2598-2606.
5. Guillier M, Gottesman S. 2006. Remodelling of the *Escherichia coli* outer membrane by two small regulatory RNAs. Mol Microbiol 59:231-247.
6. Beisel CL, Storz G. 2011. The base-pairing RNA spot 42 participates in a multioutput feedforward loop to help enact catabolite repression in *Escherichia coli*. Mol Cell 41:286-297.
